# Early-Life Ozone Exposure and Asthma and Wheeze in Children

**DOI:** 10.1001/jamanetworkopen.2025.4121

**Published:** 2025-04-02

**Authors:** Logan C. Dearborn, Marnie F. Hazlehurst, Allison R. Sherris, Adam A. Szpiro, Drew B. Day, Christine T. Loftus, Magali N. Blanco, Margaret A. Adgent, Aileen R. Andrade-Torres, Yu Ni, Mary E. Crocker, Jianzhao Bi, Joel D. Kaufman, Ruby H. N. Nguyen, Kaja Z. LeWinn, Paul E. Moore, Kecia N. Carroll, Catherine J. Karr

**Affiliations:** 1 Department of Environmental and Occupational Health Sciences, School of Public Health, University of Washington, Seattle; 2 Center for Child Health, Behavior, and Development, Seattle Children’s Research Institute, Seattle, Washington; 3 Department of Biostatistics, School of Public Health, University of Washington, Seattle; 4 Department of Health Policy, Vanderbilt University Medical Center, Nashville, Tennessee; 5 Department of Epidemiology, University of Washington, Seattle; 6 School of Public Health, College of Health and Human Services, San Diego State University, San Diego, California; 7 Center for Respiratory Biology and Therapeutics, Seattle Children’s Research Institute, Seattle, Washington; 8 Division of Pulmonary and Sleep Medicine, Department of Pediatrics, University of Washington, Seattle; 9 Department of Medicine, School of Medicine, University of Washington, Seattle; 10 Division of Epidemiology and Community Health, University of Minnesota School of Public Health, Minneapolis; 11 Department of Psychiatry and Behavioral Sciences, University of California, San Francisco; 12 Division of Allergy, Immunology, and Pulmonary Medicine, Department of Pediatrics, Vanderbilt University Medical Center, Nashville, Tennessee; 13 Department of Pediatrics, Icahn School of Medicine at Mount Sinai, New York, New York; 14 Department of Environmental Medicine and Public Health, Icahn School of Medicine at Mount Sinai, New York, New York; 15 Department of Pediatrics, School of Medicine, University of Washington, Seattle

## Abstract

**Importance:**

Ozone (O_3_) is the most frequently exceeded air pollutant standard in the US. While short-term exposure is associated with acute respiratory health, the epidemiologic evidence linking postnatal O_3_ exposure to childhood asthma and wheeze is inconsistent and rarely evaluated as a mixture with other air pollutants.

**Objectives:**

To determine associations between ambient O_3_ and subsequent asthma and wheeze outcomes both independently and in mixture with fine particulate matter and nitrogen dioxide in regions with low annual O_3_ concentrations.

**Design, Setting, and Participants:**

This cohort study consisted of a pooled, multisite analysis across 6 US cities using data from the prospective ECHO-PATHWAYS consortium (2007-2023). Included children had complete airway surveys, complete address histories from age 0 to 2 years, and a full term birth (≥37 weeks). Logistic regression and bayesian kernel machine regression (BKMR) mixture analyses were adjusted for child anthropomorphic, socioeconomic, and neighborhood factors.

**Exposures:**

Exposure to ambient O_3_ in the first 2 years of life derived from a validated point-based spatiotemporal model using residential address histories.

**Main Outcomes and Measures:**

The primary outcome was asthma and wheeze at ages 4 to 6 years; the secondary outcome was asthma and wheeze at ages 8 to 9 years. Outcomes were based on caregiver reports derived from a validated survey.

**Results:**

The analytic sample of 1188 participants had a mean (SD) age of 4.5 (0.6) years at the age 4 to 6 years visit and consisted of 614 female participants (51.7%) and 663 mothers who had a bachelor’s degree or higher (55.8%). The mean (SD) O_3_ concentration was 26.1 (2.9) parts per billion (ppb). At age 4 to 6 years, 148 children had current asthma (12.3%) and 190 had current wheeze (15.8%). The odds ratio per 2 ppb higher O_3_ concentration was 1.31 (95% CI, 1.02-1.68) for current asthma and 1.30 (95% CI, 1.05-1.64) for current wheeze at age 4 to 6 years; null associations were observed for outcomes at age 8 to 9 years, and for sensitivity covariate adjustment. BKMR suggested that higher exposure to O_3_ in mixture was associated with current asthma and wheeze in early childhood.

**Conclusions and Relevance:**

In this cohort study with relatively low ambient O_3_ exposure, early-life O_3_ was associated with asthma and wheeze outcomes at age 4 to 6 years and in mixture with other air pollutants but not at age 8 to 9 years. Regulating and reducing exposure to ambient O_3_ may help reduce the significant public health burden of asthma among US children.

## Key Points

**Question:**

Is early-life ozone (O_3_) exposure associated with asthma and wheeze in children independently and within mixtures of other air pollutants?

**Findings:**

In this multisite cohort study of 1188 children, higher mean O_3_ exposure between birth and age 2 years was associated both independently and within mixtures of fine particulate matter and nitrogen dioxide with higher odds of caregiver-reported asthma and wheeze at ages 4 to 6 years but not ages 8 to 9 years.

**Meaning:**

These findings suggest that in areas of low ambient O_3_ pollution, early-life O_3_ exposure was associated with asthma and wheeze in children.

## Teaser

This cohort study evaluates associations between ambient ozone exposure from age 0 to 2 years and subsequent asthma and wheeze outcomes at 4 to 6 and 8 to 9 years among children in regions with low annual ozone concentrations.

## Introduction

Asthma is the most common chronic disease in childhood, impacting 6.5% of children living in the US in 2021.^1^ Without a cure, research on causative agents is particularly important to prevent lifelong morbidity. Exposure to fine particulate matter (PM_2.5_), nitrogen dioxide (NO_2_), and other modifiable early-life environmental exposures has been associated with the development of asthma and wheeze during childhood and provides targets for preventive efforts.^2,3,4,5^

Short-term exposure to ozone (O_3_), a criteria air pollutant regulated by the US Environmental Protection Agency, is consistently associated with acute asthma exacerbation,^6,7,8^ although the role of long-term exposure on childhood asthma and wheeze development remains unclear. Animal toxicological studies support the biological plausibility of O_3_ developmental toxicity^9,10,11,12,13^ and indicate a more pronounced effect from O_3_ exposures during early life.^10,11^ This period coincides with immense immune and respiratory development among children^14,15^ that supports a critical window for O_3_ exposures during early life. The epidemiologic literature is mixed, with some linking long-term exposure to O_3_ with asthma exacerbations^16,17,18^ or diagnosed asthma^18,19^ while others report null associations^20,21,22^ or lower risk of current asthma and wheeze.^23,24,25^ The literature lacks adequate consideration of multipollutant effects and often relies on nonstandardized windows of exposure that obscure relevant periods for chronic airway disease development.

We extend the existing literature by investigating the association of O_3_ exposure during the first 2 years of life with odds of current asthma and wheeze using a well-characterized and geographically diverse pooled prospective cohort with low annual ambient O_3_ exposures. We additionally explore the association of O_3_ in a multipollutant mixture on asthma and wheeze at age 4 to 6 years as well as examine wheezing trajectories between ages 4 to 6 years and 8 to 9 years. We hypothesized that higher exposures to ambient O_3_ in both single and multipollutant models would be associated with higher odds of asthma and wheezing phenotypes across all ages.

## Methods

### Study Population

Participants were drawn from the ECHO prenatal and early childhood pathways to health consortium (ECHO-PATHWAYS) consortium consisting of 3 prospective pediatric cohorts: The Conditions Affecting Neurocognitive Development in Early Childhood (CANDLE), the PATHWAYS Global Alliance to Prevent Prematurity and Stillbirth (PATHWAYS-GAPPS), and The Infant Development and the Environment Study (TIDES).^26^ All research activities for this analysis were approved by the University of Washington and site institutional review boards. Women provided informed consent for themselves and their children and children assented to the age 8 to 9 years study visit. This cohort study followed the Strengthening the Reporting of Observational Studies in Epidemiology (STROBE) reporting guideline.

Cohorts have been described previously.^26^ CANDLE recruited 1503 women aged 16 to 40 years and in the second trimester with singleton, low-medical-risk pregnancies within Shelby County (Memphis), Tennessee between 2006 and 2011.^27^ The GAPPS biorepository recruited pregnant women aged 18 years or older and collected demographic, health, and biospecimens between 2011 and 2016. A total of 669 participants recruited in Seattle and Yakima, Washington were recontacted and enrolled into PATHWAYS-GAPPS. TIDES recruited 900 pregnant women with low medical risk aged 18 years or older in the first trimester from 4 obstetrical clinics located in Minneapolis, Minnesota; Rochester, New York; San Francisco, California; and Seattle, Washington between 2010 and 2012.^28^

**Figure 1.**
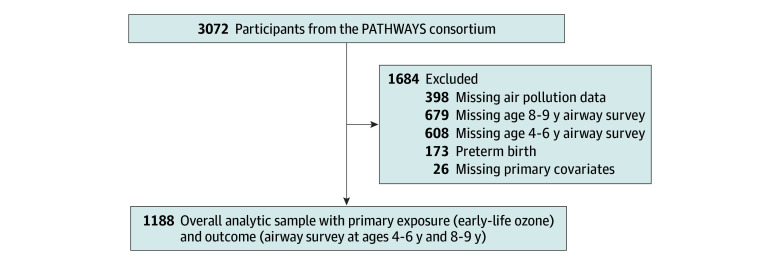
Inclusion Diagram Depicts the full potential sample in the ECHO prenatal and early childhood pathways to health consortium (ECHO-PATHWAYS) and reasons for exclusion from the analytic sample.

Eligible participants had valid geocoded address histories between birth and age 2 years, a completed airway survey at both ages 4 to 6 years and 8 to 9 years, and were not missing primary model covariates (Figure 1). Preterm births (<37 weeks) were excluded due to higher rates of airway disease among this population.

### Exposures

Early-life O_3_ exposures (parts per billion [ppb]) were estimated by averaging the concentrations from a validated, point-based national spatiotemporal model between birth and age 2 years described elsewhere in detail.^29,30,31,32^ In brief, the model uses pollutant concentrations and hundreds of geographic covariates from regulatory agency monitors and research campaigns, reduced via partial least squares. A spatiotemporal generalization of universal kriging applied to observed time trends is used to predict point-based temporally resolved estimates of ambient outdoor pollutant concentrations. Model performance was good, with a cross-validated R^2^ of 0.737 for long-term spatial estimates and 0.834 for long-term temporal estimates. Biweekly averages constructed from 24-hour mean pollutant concentrations were estimated at all residential locations for each child between birth and age 2 years, representing a 2-year estimate. The same method and averaging period were used for NO_2_ (ppb) and PM_2.5_ (μg/m^3^) for multipollutant models.^29,30,32^ Prenatal pollutant averages (conception through birth) were covariates in a sensitivity analysis.

### Outcomes

Caregivers completed airway surveys at age 4 to 6 year- and 8 to 9 year-visits in all cohorts using the International Study of Asthma and Allergies in Childhood (ISAAC), described previously.^33,34,35^ Primary outcomes were ascertained at age 4 to 6 years and included current wheeze defined as endorsement of “Has the child had wheezing or whistling in the chest in the past 12 months?,” while current asthma was defined as endorsement of at least 2 of the 3 criteria: “Has your child ever had asthma?,” “Does the child use any medications for treatment of recurrent cough, recurrent wheezing, or asthma?,” and/or meets current wheeze definition.

In secondary analyses, asthma at age 8 to 9 years, strict asthma, required caregiver report of physician-diagnosed asthma and an affirmative answer to current asthma medication use or current wheeze at age 8 to 9 years. Wheezing trajectories between age 4 to 6 years and 8 to 9 years were defined as early wheeze (report of ever wheeze at age 4 to 6 years but not current wheeze at age 8 to 9 years), late wheeze (those who reported ever wheeze at age 8 to 9 years but not at age 4 to 6 years), persistent wheeze (those reporting ever wheeze at age 4 to 6 years and current wheeze at age 8 to 9 years), and never wheeze (those with a response of no to wheeze at both time points), while those who indicated “don’t know” at either time point were excluded. Analyses of outcomes ascertained at age 8 to 9 years were considered secondary due to anticipating more proximal exposures being relevant for the development of airway disease.^4,33,34^

### Covariates

We selected covariates a priori, informed by literature on known and proxies for risk factors or precision variables. Child factors included sex assigned at birth (male or female), child age at assessment (continuous), year of birth (splines with 1 degree of freedom per year), postnatal secondhand smoke exposure (SHS) (binary), breastfeeding duration (never, <6 months, or ≥6 months), and furry pet ownership in the first year of life (binary). Maternal and household factors included education at the age 4 to 6 years visit (less than high school, high school, some college, or 4-year college degree or postgraduate degree), maternal history of asthma (binary), region-and-inflation adjusted household income at age 4 to 6 years (USD), household size at age 4 to 6 years (<4, 4, 5, or >5), maternal prenatal smoking (combined self-report and/or midpregnancy cotinine concentration >200 ng, binary), and neighborhood deprivation index (NDI) averaged over the first 2 years of life.^36,37^ Recruitment site was included to address unmeasured confounding.

### Statistical Analysis

Descriptive analyses were used to summarize characteristics of the study population. Logistic regression with staged covariate modeling assessed odds ratios (ORs) and 95% CIs of approximate IQR higher early-life O_3_ (birth to age 2 years) exposure (2 ppb) on asthma and wheeze outcomes at age 4 to 6 years using robust SEs.^38^ A minimal model adjusted for child sex, age, birth year, and site. A primary model additionally adjusted for maternal education, maternal asthma history, postnatal SHS exposure, and NDI. An extended model additionally adjusted for precision variables and those thought to be colinear with variables included in the primary model: household income interacted with household size, furry pet ownership, maternal prenatal smoking, and duration of breastfeeding.

Sensitivity analyses included adjustment for prenatal O_3_, NO_2,_ and PM_2.5_ as well as postnatal NO_2_ and PM_2.5_ on the association between early-life O_3_ and asthma and wheeze. Leave-1-cohort-and-site analyses systematically excluded a single cohort or site. Logistic general additive models (GAMs) were used to explore nonlinear associations of O_3_ exposure on current asthma and wheeze. All sensitivity analyses used the primary covariate adjustment model.

Probit bayesian kernel machine regression (BKMR)^39,40^ was used to explore health outcomes of O_3_ after the adjustment and interaction of postnatal NO_2_ and PM_2.5_ concentrations as well as explore the overall mixture on asthma and wheeze at age 4 to 6 years. Model estimates represent probability *z* scores of a given outcome. The model was fit using Markov Chain Monte Carlo for 50 000 iterations in 4 parallel chains, with the first 12 000 used as a burn-in period.

Secondary analyses used logistic regression to explore early-life O_3_ on strict asthma at age 8 to 9 years. Multinomial regression was used to assess wheezing phenotypes with never wheeze serving as the reference population. Secondary analyses adjusted for primary covariates with child age reflecting the age 8 to 9 years visit. All analyses were performed in R 4.2.2 (R Foundation for Statistical Computing).

## Results

A total of 1188 participants met eligibility requirements with 745 (62.7%) being from CANDLE, 133 (11.2%) from PATHWAYS-GAPPS, and 310 (26.1%) from TIDES. Their mean (SD) age was 4.5 (0.6) years at the age 4 to 6 years visit, 614 (51.7%) were female, and 973 (81.9%) had mothers without a history of asthma (Table 1). Participant characteristics among those excluded from the present analysis can be found in eTable 1 in Supplement 1. At the age 4 to 6 years visit, 148 participants (12.3%) had current asthma and 190 (15.8%) had current wheeze. At the age 8 to 9 years visit, the mean (SD) age was 8.9 (0.7) years, 113 (9.4%) had strict asthma and, using information from both airway assessments, 715 (59.5%) were classified as never wheeze, 250 (20.8%) early wheeze, 136 (11.3%) late wheeze, and 100 (8.3%) persistent wheeze.

**Table 1.** Characteristics of the Analytic Sample

Characteristic	Participants, No. (%) (n = 1188)^a^
Cohort and site	
CANDLE	745 (62.7)
GAPPS	133 (11.2)
Seattle-GAPPS, Washington	66 (5.6)
Yakima, Washington	67 (5.6)
TIDES	310 (26.1)
Minneapolis, Minnesota	100 (8.4)
Rochester, New York	73 (6.1)
San Francisco, California	65 (5.5)
Seattle-TIDES, Washington	72 (6.1)
Maternal education at age 4-6 y	
<High school	45 (3.8)
High school or equivalent	316 (26.6)
Some college or technical school	164 (13.8)
Bachelor’s degree or higher	663 (55.8)
Maternal history of asthma	215 (18.1)
Prenatal smoking (19 missing)^b^	103 (8.7)
Child sex assigned at birth	
Female	614 (51.7)
Male	574 (48.3)
Age at 4-6 y visit, mean (SD), y	4.5 (0.6)
Age at 8-9 y visit, mean (SD), y	8.9 (0.7)
Breastfeeding duration (7 missing)	
None	266 (22.4)
<6 mo	720 (60.6)
≥6 mo	195 (16.4)
Year of birth	
2007	49 (4.1)
2008	118 (9.9)
2009	179 (15.1)
2010	208 (17.5)
2011	303 (25.5)
2012	223 (18.8)
2013	76 (6.4)
2014	27 (2.3)
2015	5 (0.4)
Secondhand smoke exposure at 4-6 y	233 (19.6)
Neighborhood Deprivation Index birth-age 2 y, mean (SD)	0.1 (0.78)
Income at age 4-6, mean (SD), USD (36 missing)^c^	66 938 (54 302)
Household size age 4-6 y (34 missing)	
<4	233 (19.6)
4	477 (40.2)
5	256 (21.5)
≥5	188 (15.8)
Furry pets in the first 12 mo of life (2 missing)	565 (47.6)
Current asthma, age 4-6 y^d^	148 (12.3)
Current wheeze, age 4-6 y^e^	190 (15.8)
Age 8-9 y strict asthma^f^	113 (9.4)
Wheezing trajectories, age 4-6 y and 8-9 y^g^	
Never	715 (59.5)
Early	250 (20.8)
Late	136 (11.3)
Persistent	100 (8.3)

^a^ Participants must have attended both the age 4 to 6 years and the age 8 to 9 years study visits to be included in the present analysis.

^b^ Combined self-report and/or midpregnancy cotinine 200 mg/dl or higher.

^c^ Region and inflation adjusted household income.

^d^ Current asthma (age 4-6 years) defined as affirmative to “Has your child ever had asthma,” and/or “Has the child had wheezing or whistling in the chest in the past 12 months?,” and/or “Does the child use any medications for treatment of recurrent cough, recurrent wheezing, or asthma?”

^e^ Current wheeze defined as yes to “Has the child had wheezing or whistling in the chest in the past 12 months?”

^f^ Age 8 to 9 years strict asthma defined as affirmative to “Have you ever been told by a physician or other health care clinician that the child has asthma or reactive airway disease?, and “Has the child had wheezing or whistling in the chest in the past 12 months?,” and/or “Does the child use any medications for treatment of recurrent cough, recurrent wheezing, or asthma?”

^g^ Wheezing trajectories defined as early wheeze only at age 4 to 6 years, late wheeze only at age 8 to 9 years, persistent wheeze at both age 4 to 6 and 8 to 9 years, and never report of wheeze.

The mean (SD) ambient O_3_ concentration between birth and age 2 years was 26.1 (2.9) ppb (Table 2). See eFigure 1 in Supplement 1 for site-specific O_3_ distributions. Additional postnatal pollutant means (SD) were 8.8 (2.7) ppb NO_2_ and 9.3 (1.8) μg/m^3^ PM_2.5_. O_3_ was negatively correlated with NO_2_ (Pearson correlation: −0.46) and positively with PM_2.5_ (0.48) in the overall sample.

**Table 2.** Air Pollutant Exposure Distributions

Pollutant and Window	Mean (SD)	Distribution, ppb				
		10%	25%	50%	75%	90%
O_3_						
0-2 y	26.1 (2.9)	20.8	25.3	26.6	27.8	29.0
Prenatal	25.6 (3.5)	21.1	23.8	26.0	28.0	29.8
NO_2_						
0-2 y	8.8 (2.7)	5.2	7.0	8.8	10.5	12.1
Prenatal	8.7 (3.1)	4.9	6.5	8.4	10.7	12.7
PM_2.5_, μg/m^3^						
0-2 y	9.3 (1.8)	6.2	8.2	9.8	10.6	11.0
Prenatal	9.8 (2.1)	6.4	8.6	10.2	11.3	12.0

Abbreviations: NO_2_, nitrogen dioxide; O_3_, ozone; PM_2.5_, fine particulate matter; ppb, parts per billion.

**Figure 2.**
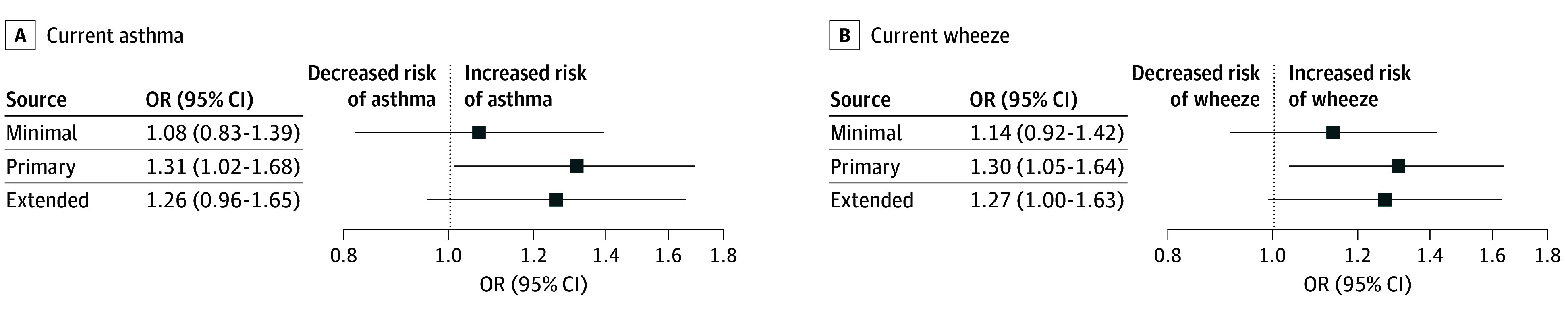
Association Between Early-Life Ozone (O_3_) and Current Asthma and Wheeze at Age 4 to 6 Years Odds ratios (ORs) and 95% CIs of 2 ppb higher early-life O_3_ are depicted. Minimal models were adjusted for child sex, age, birth year, and site. Primary models were additionally adjusted for maternal education, maternal asthma status, postnatal secondhand smoke exposure, and Neighborhood Deprivation Index. Extended models were additionally adjusted for household income interacted with household size, furry pet ownership, maternal prenatal smoking, and duration of breastfeeding.

Using primary covariate adjustment, 2 ppb higher early-life O_3_ was associated with an OR of 1.31 (95% CI, 1.02-1.68) for current asthma and 1.30 (95% CI, 1.05-1.64) for current wheeze at the age 4 to 6 years visit (Figure 2). Both minimal and, to a lesser extent, extended covariate adjustment attenuated estimates compared with the primary model; ORs were 1.08 (95% CI, 0.83-1.39) and 1.26 (95% CI, 0.96-1.65) for current asthma, respectively, and 1.14 (95% CI, 0.92-1.42) and 1.27 (95% CI, 1.00-1.63) for current wheeze, respectively.

Compared with the primary findings, prenatal and copollutant adjustment attenuated associations between early-life O_3_ and airway outcomes so that the findings were no longer statistically significant (eFigure 2 in Supplement 1). Estimates when a cohort or site was omitted were relatively consistent apart from the exclusion of CANDLE that attenuated findings (eFigure 3 in Supplement 1). GAMs suggested that the association between O_3_ and current asthma leveled off at about 27 ppb O_3_; there was no evidence of a nonlinear association for current wheeze (eFigure 4 in Supplement 1).

**Figure 3.**
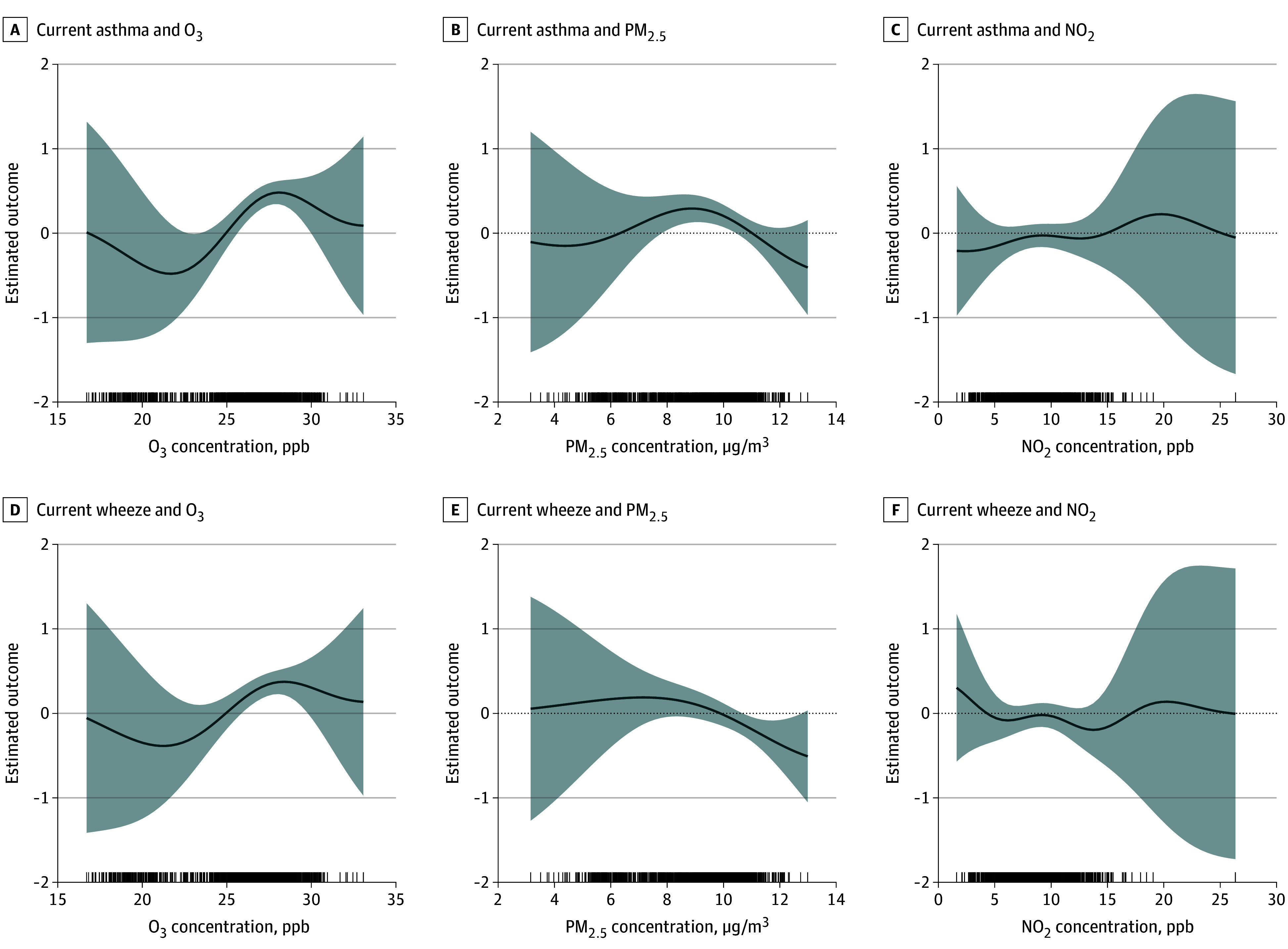
Probit Bayesian Kernel Machine Regression Exposure-Response Function of Each Pollutant When Other Pollutants Are Held to Their Median Value This figure represents the change in the predicted outcome probability *z* score as 1 pollutant increases in concentration while the other 2 pollutants are held to their median. A-C, Current asthma at age 4 to 6 years, and D-F, current wheeze at age 4 to 6 years. A rug plot depicting pollutant concentrations can be found along the x-axis. NO_2_ indicates nitrogen dioxide; O_3_, ozone; PM_2.5_, fine particulate matter; ppb, parts per billion.

There was a suggestion of higher asthma risk as the combined O3, NO2, and PM2.5 mixture increased up to its median level using BKMR (eFigure 5 in Supplement 1). We observed no association between the exposure mixture and current wheeze. When both NO_2_ and PM_2.5_ were held to their median values, there was a linear association between higher exposure to O_3_ and both current asthma and wheeze between concentrations of approximately 25 to 28 ppb (Figure 3). Bivariate interactions depicted consistent associations between O_3_ and current asthma across all concentrations of NO_2_ (eFigure 6 in Supplement 1). The evidence for associations between O_3_ and current asthma and wheeze were consistent for PM_2.5_ held at the 50th or 90th percentile but were null for lower levels of PM_2.5_ (eFigures 6 and 7 in Supplement 1). Bivariate analysis of O_3_ and current wheeze showed limited associations across quantiles of NO_2_.

Secondary analyses were null; the OR for age 8 to 9 years strict asthma was 1.06 (95% CI, 0.77-1.45), while the OR was 1.00 (95% CI, 0.89-1.43) for early wheeze, 0.96 (95% CI, 0.86-1.57) for late wheeze and 1.16 (95% CI, 0.84-1.73) for persistent wheeze when compared with the reference of never wheeze (eTable 2 in Supplement 1).

## Discussion

Higher ambient O_3_ in the first 2 years of life was associated with both current asthma and wheeze at age 4 to 6 years in this pooled, multisite analysis in this relatively low O_3_ environment. Within mixture, higher O_3_ was consistently associated with higher asthma and wheeze when other pollutants were held at the median, although pairwise associations differed by pollutant. No association was observed between early-life O_3_ and age 8 to 9 years strict asthma, nor for wheezing trajectories between ages 4 to 6 years and 8 to 9 years.

Chronic exposure to O_3_ in early life has well-demonstrated pulmonary impacts in animal models that are thought to progress the development of asthma and wheeze in children.^41^ Key mechanisms include disruption of airway remodeling through O_3_ induced inflammatory pathways^9^ as well as interrupted alveolar morphogenesis from chronic O_3_ exposure in infancy.^11,12^ Additionally, cyclical and episodic O_3_ exposure during infancy caused altered pulmonary morphology and structure in nonhuman primates.^13^ Induction of oxidative stress^10^ as well as pulmonary cell proliferation^42^ and differential gene expression related to cell motility and branching morphogensis^43^ have also been observed after exposure to O_3_ in the lungs of neonate rats. Taken together, evidence in animal models support the biologic plausibility of chronic early-life exposure to O_3_ influencing the development of asthma in humans.

Understanding the influence of postnatal O_3_ on development of pediatric asthma is important as it represents the criteria air pollutant most commonly exceeded among US children.^44^ Average ambient exposures were lower in our study than those reported in other studies of O_3_ and childhood asthma.^16,17,18,19,21,22,24,25^ We observed higher odds of current asthma and wheeze at age 4 to 6 years with higher early-life O_3_ exposure in a community with modest ambient O_3_ concentrations. However, these associations did not persist in all sensitivity analyses, particularly for the current wheeze outcome when the CANDLE cohort was excluded, which may reflect a loss in power or cohort-specific bias from unmeasured confounding. In general, sensitivity analyses led to widened CIs for asthma with a relatively consistent effect estimate, which may indicate greater stability in this outcome compared with current wheeze. Examination of a persistent wheeze phenotype based on reports at both age 4 to 6 years and 8 to 9 years did not show a significant association. Null findings at this older age may indicate that airway outcome development is related to more proximal ambient O_3_ concentrations, although more research is needed in this area. While several studies have examined chronic O_3_ exposures in pediatric epidemiologic analyses of asthma,^16,17,18,19,20,21,22,23,24,25^ a consistent message has not emerged from this body of work. This likely reflects the large diversity of study designs regarding childhood exposure windows and concentrations as well as outcome ages and definitions.

We emphasized exposure during a critical period of development^14,15^ and asthma at ages when diagnostic certainty is greater (above 4 years) and into school age (8-9 years) when current asthma and persistent wheeze may reflect a more sustained airway disease.^45^ Among prior studies, a small number investigated this early exposure window and child asthma assessment beyond the preschool period. To et al^22^ conducted a large administrative cohort in Ontario, Canada, reporting a suggested effect of O_3_ averaged between ages 0 to 3 years on asthma-related health care utilization at 5 to 9 years. After adjustment for NO_2_, PM_2.5_, and normalized difference vegetation index, they report a hazard ratio (HR) of 1.13 (95% CI, 0.97-1.31) compared with an estimate of 1.02 (95% CI, 0.90-1.14) in O_3_ only models. Nishimura et al^21^ conducted a case-control study among African American and Latino children recruited in urban areas across the US and Puerto Rico aged 8 to 21 years. No association was observed between daily maximum O_3_ exposure averaged over ages 0 to 3 years and physician-diagnosed asthma with symptoms in the past 2 years (OR, 0.98; 95% CI, 0.84-1.13).

It is a widely recognized need to better understand the health effects of O_3_ and other air pollutants in the context of pollutant mixtures.^46^ While our copollutant-adjusted regressions were attenuated, our BKMR findings suggest O_3_ contributed to higher asthma at and above median concentrations of NO_2_ and PM_2.5_, indicating possible effect modification by these other criteria pollutants. A post hoc GAM additionally identified a potential leveling off for O_3_ above 27 ppb in single pollutant models for current asthma. Understanding the shape of both single and multipollutant exposure-response functions is an area where more research is needed. In the only analysis of O_3_ mixtures and childhood asthma and wheeze that we identified, Tian et al^47^ found an overall mixture association for combined asthma and wheeze with a mean age of 3.2 years with age 0 to 1 year exposure to O_3_, NO_2_, sulfur dioxide (SO_2_), PM_2.5_, and carbon monoxide when using quantile-based g-computation; they estimated a mixture HR of 1.65 (95% CI, 1.30-2.10) associated with SO_2_, NO_2_, and minorly O_3_. In contrast, the same analysis found an opposite effect from O_3_ in the 0 to 2 years window where it was weighted negatively, finding an HR of 2.53 (95% CI, 2.16-2.97) associated with SO_2_ and NO_2_. Differences in the studied pollutants, geographies, and age at outcome assessment may be responsible for the disparate role of O_3_ within mixture averaged over ages 0 to 2 years between the 2 studies.

### Strengths and Limitations

This analysis has several strengths. Use of a well-characterized, multisite population with geographic and socioeconomic diversity allowed robust control for confounding and enhances generalizability of results. Additionally, we used a fine-scale spatiotemporal model to estimate ambient O_3_ concentrations at participant residences, reducing exposure misclassification compared with previous work relying on spatial-only measures. We additionally explored associations with standardized exposure and outcome windows defined on biologically relevant periods that offer insight on potentially vulnerable windows of exposure. Finally, this analysis contributes to the limited literature regarding ambient air pollutant mixtures on asthma and wheeze development in early childhood.

This analysis has several important limitations. While the use of spatiotemporal derived O_3_ estimates is an improvement within the field, no personal or indoor measurements were available. However, assessing ambient concentrations is informative for policy and regulation of outdoor pollutants. Outcomes were derived from the widely-used ISAAC survey^48^ but relied on caregiver report. There is the potential for unmeasured confounding from the indoor environment as well as incomplete adjustment of spatially-varying socioeconomic and ambient environmental factors that may have biased estimates.

## Conclusions

In conclusion, estimated exposure to O_3_ in the first 2 years of life was associated with asthma and wheeze at age 4 to 6 years but not age 8 to 9 years in a multisite study of low ambient O_3_ concentrations. Higher O_3_ was indicated within mixture to influence asthma and wheeze, although limited overall mixture associations were determined. This analysis underscores the importance of better understanding the role of early-life exposure to ambient O_3_ in addressing risk factors for pediatric asthma in the US.

## Notes

Supplement 1. **eTable 1.** Characteristics of the analytic sample and excluded participants **eTable 2.** Associations between early-life O3 and secondary airway outcomes between ages 4 to 6 and 8 to 9 years **eFigure 1. **Density of ozone (ppb) concentrations by site **eFigure 2.** Copollutant adjustment **eFigure 3. **Leave 1 cohort and site out sensitivity analysis **eFigure 4.** General additive model exploring nonlinearity **eFigure 5.** Overall mixture association when pollutant mixture was held at various quantiles **eFigure 6. **Response function for current asthma outcome exploring bivariate pollutant interactions **eFigure 7.** Response function for the current wheeze outcome exploring bivariate pollutant interactions

Supplement 2.
Data Sharing Statement
