# Supplementary material for: Early-Life Ozone Exposure and Asthma and Wheeze in Children
Source: JAMA Netw Open. 2025 Apr 2;8(4):e254121. doi: 10.1001/jamanetworkopen.2025.4121 (PMC11966328; doi:10.1001/jamanetworkopen.2025.4121)
Supplement: Supplement 1. — eTable 1. Characteristics of the analytic sample and excluded participants eTable 2. Associations between early-life O3 and secondary airway outcomes between ages 4 to 6 and 8 to 9 years eFigure 1. Density of ozone (ppb) concentrations by site eFigure 2. Copollutant adjustment eFigure 3. Leave 1 cohort and site out sensitivity analysis eFigure 4. General additive model exploring nonlinearity eFigure 5. Overall mixture association when pollutant mixture was held at various quantiles eFigure 6. Response function for current asthma outcome exploring bivariate pollutant interactions eFigure 7. Response function for the current wheeze outcome exploring bivariate pollutant interactions [file jamanetwopen-e254121-s001.pdf]

Supplemental Online Content

Dearborn LC, Hazlehurst MF, Sherris AR, et al. Early-life ozone exposure and asthma and wheeze in children. *JAMA Netw Open*. 2025;8(4):e254121.
doi:10.1001/jamanetworkopen.2025.4121

eTable 1. Characteristics of the analytic sample and excluded participants

eTable 2. Associations between early-life O3 and secondary airways outcomes between ages 4 to 6 and 8 to 9 years

eFigure 1. Density of ozone (ppb) concentrations by site

eFigure 2. Copollutant adjustment

eFigure 3. Leave 1 cohort and site out sensitivity analysis

eFigure 4. General additive model exploring nonlinearity

eFigure 5. Overall mixture association when pollutant mixture was held at various quantiles

eFigure 6. Response function for current asthma outcome exploring bivariate pollutant interactions

eFigure 7. Response function for the current wheeze outcome exploring bivariate pollutant interactions

This supplemental material has been provided by the authors to give readers additional information about their work.

© 2025 Dearborn LC et al. *JAMA Network Open*.

eTable 1. Characteristics of the analytic sample and excluded participants

Characteristic	Participants (n=1188)	Excluded (n=1884)
Cohort and site		
CANDLE	745 (62.7)	758 (40.2)
GAPPS	133 (11.2)	536 (28.5)
Seattle-GAPPS, WA	66 (5.6)	278 (14.8)
Yakima, WA	67 (5.6)	258 (13.7)
TIDES	310 (26.1)	590 (31.3)
Minneapolis, MN	100 (8.4)	133 (7.1)
Rochester, NY	73 (6.1)	177 (9.4)
San Francisco, CA	65 (5.5)	157 (8.3)
Seattle-TIDES, WA	72 (6.1)	123 (6.5)
Maternal education age 4-6		
< High school	45 (3.8)	44 (2.3)
High school or equivalent	316 (26.6)	248 (13.2)
Some college or technical school	164 (13.8)	212 (11.3)
Bachelor's degree or higher	663 (55.8)	609 (32.3)
Missing	0 (0)	771 (40.9)
Maternal education at enrollment[a]		
< High school	91 (7.7)	185 (9.8)
High school or equivalent	405 (34.1)	651 (34.6)
Some college or technical school	390 (32.8)	615 (32.7)
Bachelor's degree or higher	297 (25.0)	416 (22.1)
Missing	5 (0.4)	17 (0.9)
Maternal history of asthma		
Yes	215 (18.1)	215 (11.4)
Missing	0 (0)	775 (41.1)
Prenatal smoking		
Yes	103 (8.7)	203 (10.8)
Missing	19 (1.6)	491 (26.1)
Child sex assigned at birth		
Female	614 (51.7)	842 (44.7)
Male	574 (48.3)	905 (48.0)
Missing	0 (0)	137 (7.3)
Breastfeeding duration		
None	266 (22.4)	222 (11.8)
<6 months	720 (60.6)	562 (29.8)
≥6 months	195 (16.4)	208 (11.0)
Missing	7 (0.6)	892 (47.3)
Year of birth		

© 2025 Dearborn LC et al. *JAMA Network Open*.

2007	49 (4.1)	87 (4.6)
2008	118 (9.9)	171 (9.1)
2009	179 (15.1)	148 (7.9)
2010	208 (17.5)	164 (8.7)
2011	303 (25.5)	364 (19.3)
2012	223 (18.8)	370 (19.6)
2013	76 (6.4)	133 (7.1)
2014	27 (2.3)	89 (4.7)
2015	5 (0.4)	87 (4.6)
2016	0 (0)	89 (4.7)
2017	0 (0)	26 (1.4)
Missing	0 (0)	156 (8.3)
Secondhand smoke exposure at 4-6		
Yes	233 (19.6)	188 (10.0)
Missing	0 (0)	821 (43.6)
Neighborhood Deprivation Index birth - age 2, mean (SD)	0.1 (0.8)	0.1 (0.8)
Missing	0 (0)	382 (20.3)
Income at age 4-6 (USD), mean (SD)	66938 (54302)	73459 (57314)
Missing	36 (3.0)	898 (47.7)
Household size age 4-6		
<4	233 (19.6)	235 (12.5)
4	477 (40.2)	417 (22.1)
5	256 (21.5)	235 (12.5)
5+	188 (15.8)	188 (10.0)
Missing	34 (2.9)	758 (40.2)
Furry pets in the first 12 months of life,		
Yes	565 (47.6)	412 (21.9)
Missing	2 (0.2)	1152 (61.1)

[a]Not used in analysis

eTable 2. Associations between early-life O_3 and secondary airways outcomes between ages 4 to 6 and 8 to 9 years

Outcome	OR (95% CI)
Age 8 strict asthma	1.06 (0.77-1.45)
Wheezing trajectories	
Early	1.00 (0.89-1.43)
Late	0.96 (0.86-1.57)
Persistent	1.16 (0.84-1.73)

OR: odds ratio; 95% CI: 95% confidence interval

© 2025 Dearborn LC et al. *JAMA Network Open*.

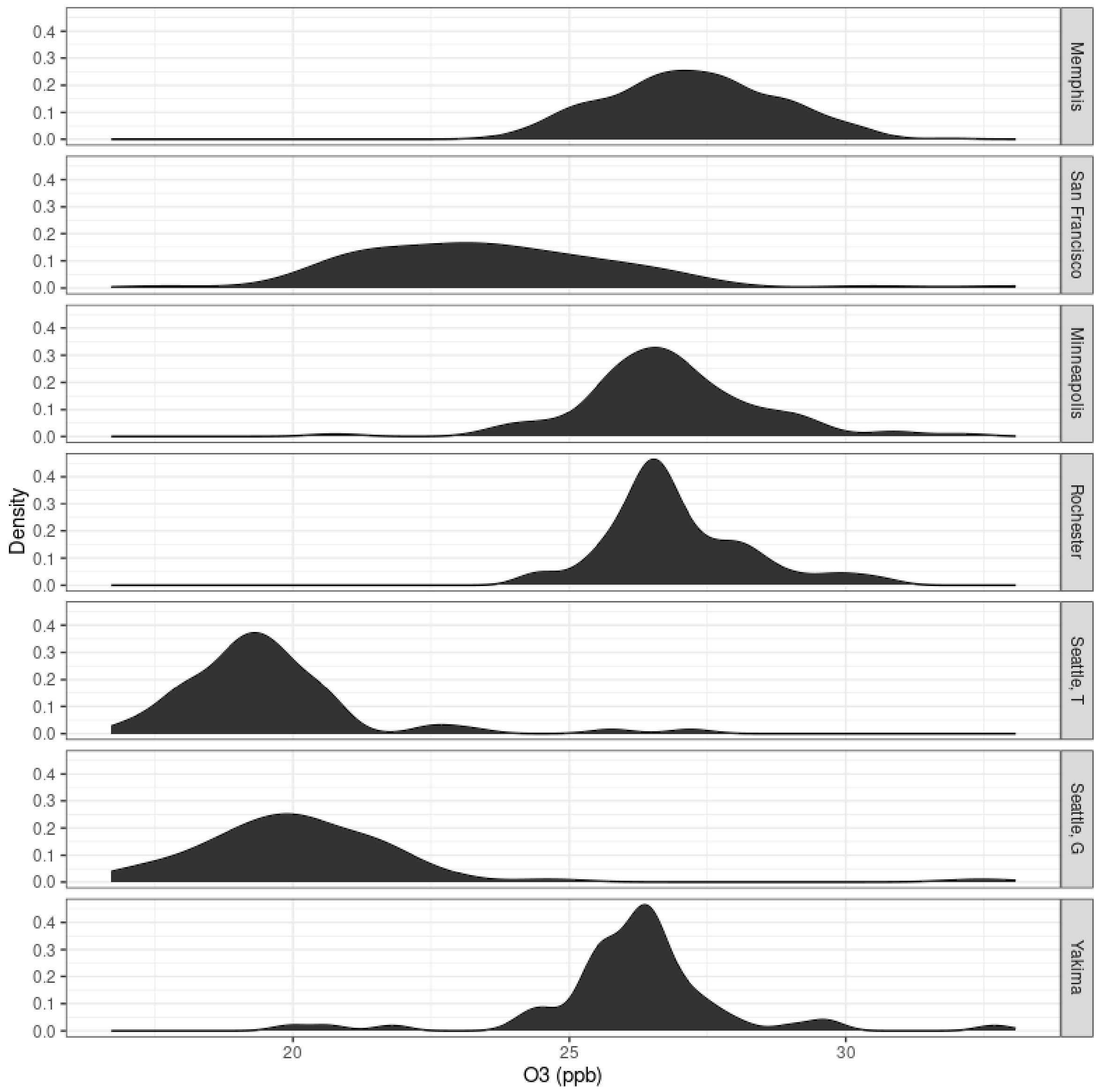

eFigure 1. Density of ozone (ppb) concentrations by site. Ozone concentrations were estimated using the participants residential history between birth and age 2, representing the two-year long-term average. Seattle, T and Seattle, G represent the concentrations at the Seattle-TIDES and Seattle-GAPPS sites, respectively.

© 2025 Dearborn LC et al. *JAMA Network Open*.

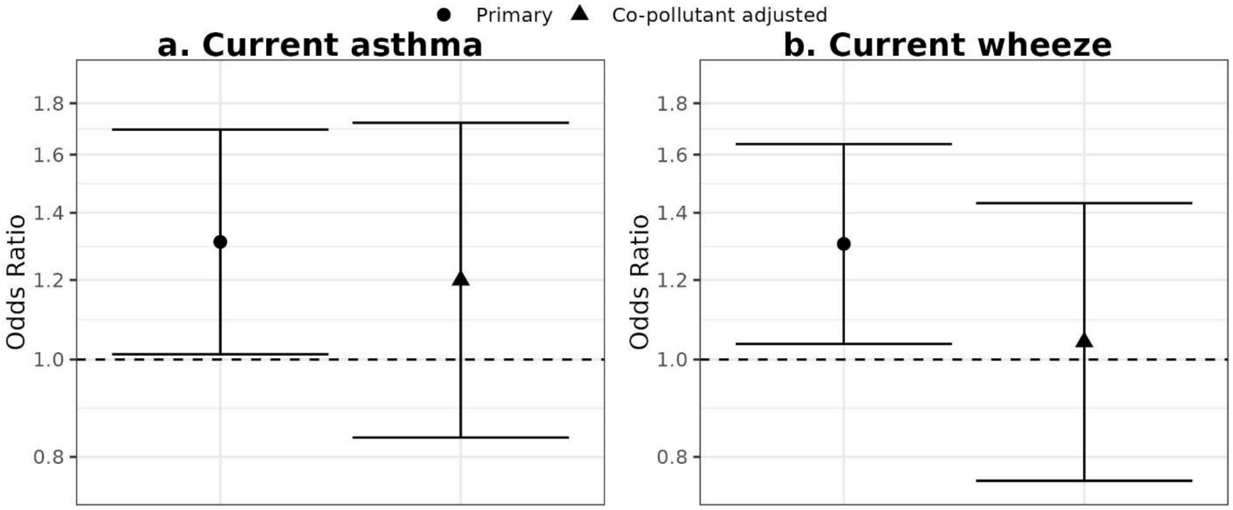

eFigure 2. Copollutant adjustment. Prenatal averages of NO_2, O_3, and $PM_{2.5}$ as well as postnatal age 0–2-year averages of NO_2 and $PM_{2.5}$ were adjusted in logistic regressions using the primary covariate staging: child sex, age, birth year, site, maternal education, maternal asthma status, postnatal secondhand smoke exposure, and NDI. The postnatal O_3 odds ratio and 95% confidence interval are depicted.

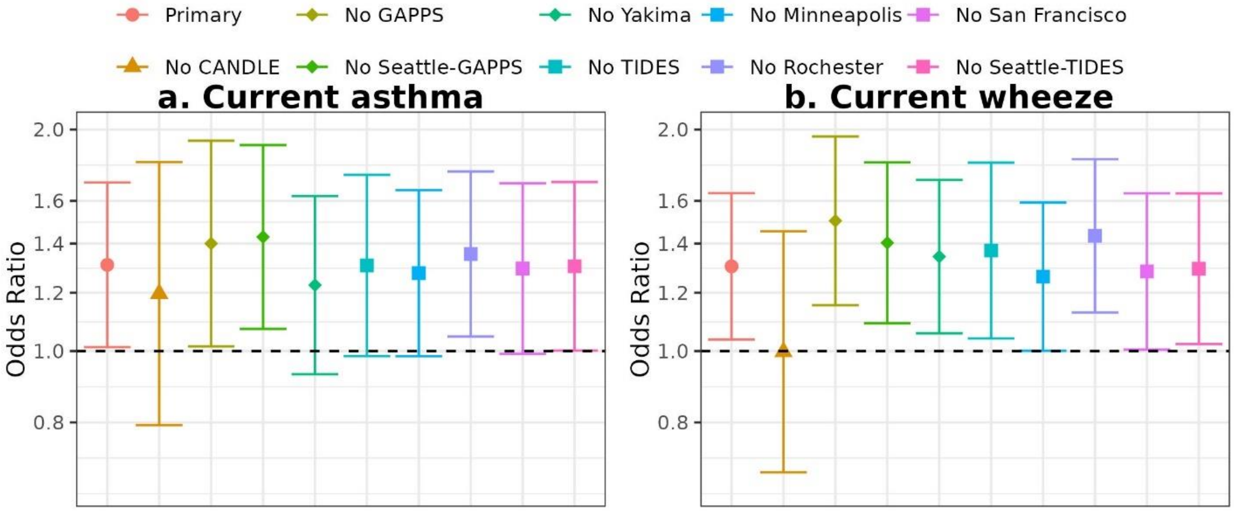

eFigure 3. Leave 1 cohort and site out sensitivity analysis. Each cohort and site were systematically excluded from a logistic regression using the primary covariate staging: child sex, age, birth year, site, maternal education, maternal asthma status, postnatal secondhand smoke exposure, and NDI. The shape of the estimate represents the cohort where each site is affiliated; the CANDLE cohort is represented by triangles, the GAPPS cohort and sites are represented by diamonds, and the TIDES cohort and sites are represented by squares.

© 2025 Dearborn LC et al. *JAMA Network Open*.

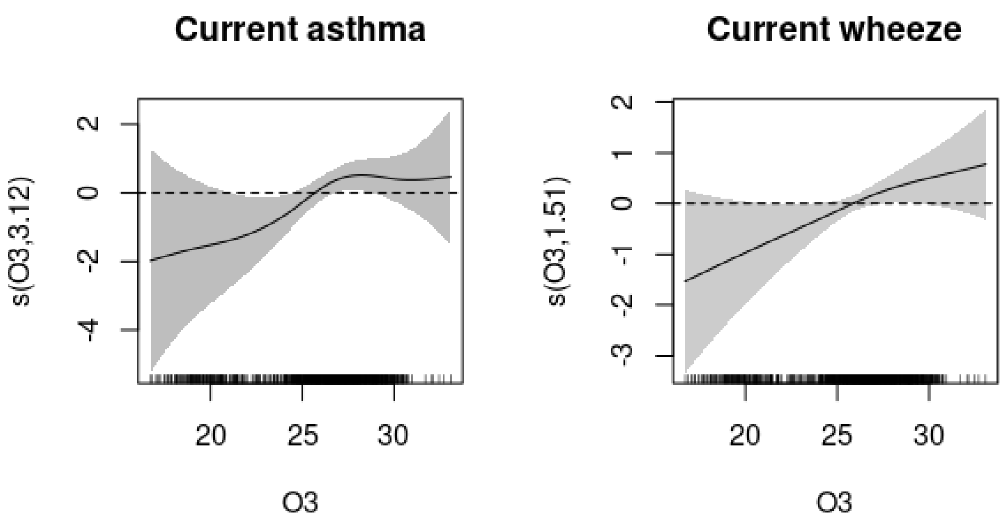

eFigure 4. General additive model exploring nonlinearity

Logistic Generalized Additive Models (GAMs) were used to visually assess for non-linearity of exposure-response functions between early-life O_3 exposure and asthma and wheeze at age 4-6. GAMs were adjusted using primary covariate staging: child sex, age, birth year, site, maternal education, maternal asthma status, postnatal secondhand smoke exposure, and neighborhood deprivation index.

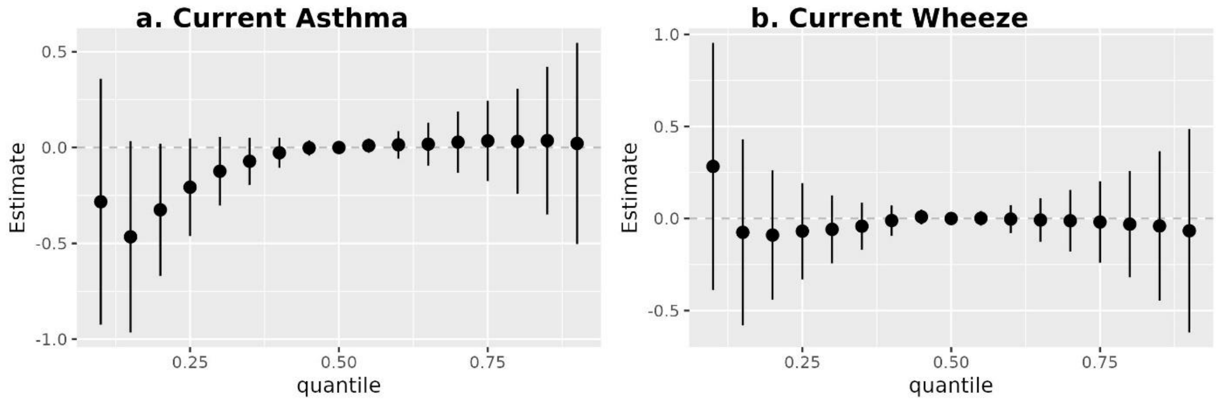

eFigure 5. Overall mixture association when pollutant mixture was held at various quantiles

The probit BKMR-estimated outcome probability z-score associated with the air pollutant mixture when each pollutant was set to the same quantile value. These estimates are zeroed at the median exposure concentration for all pollutants.

© 2025 Dearborn LC et al. *JAMA Network Open*.

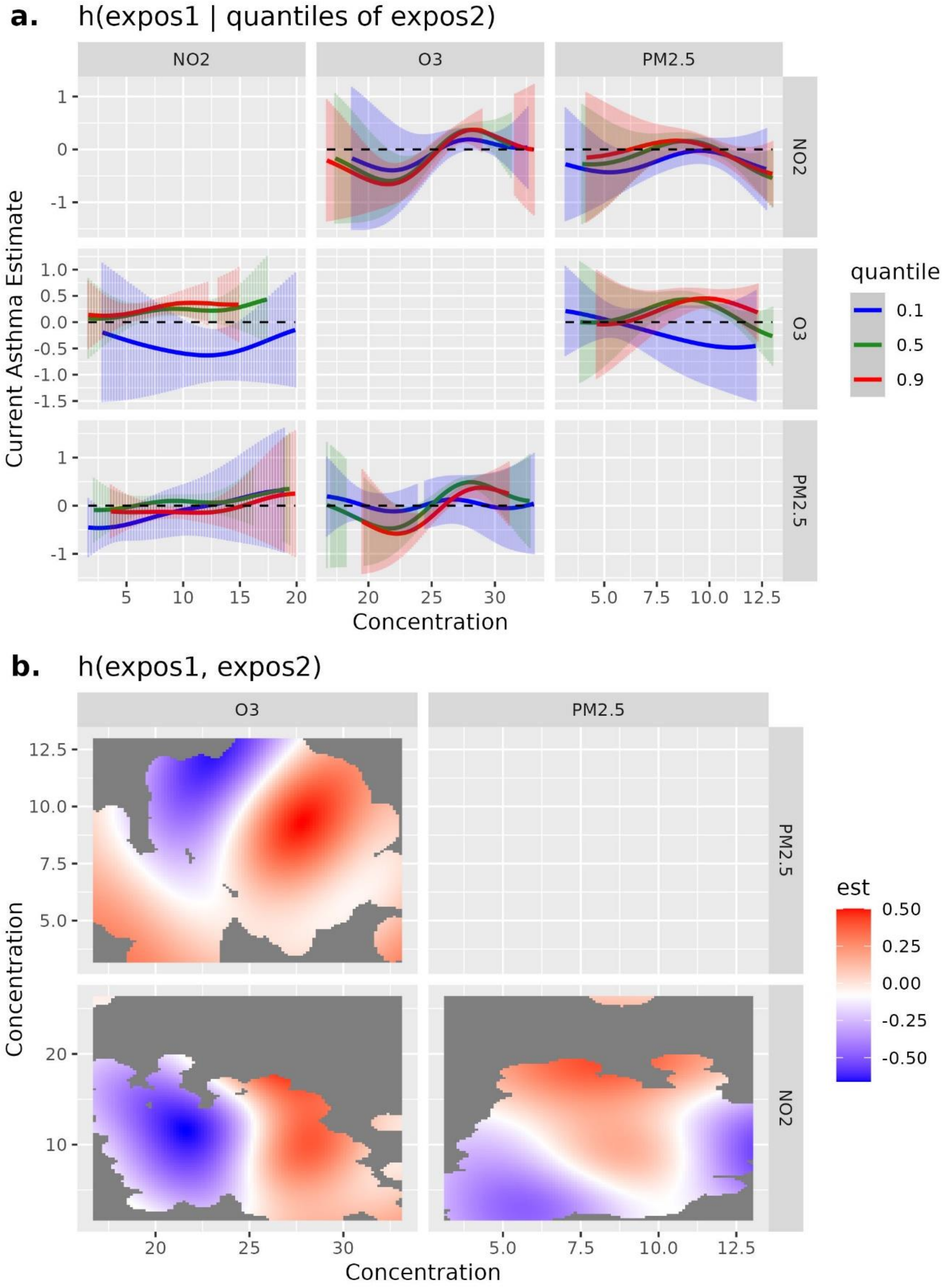

eFigure 6. Response function for current asthma outcome exploring bivariate pollutant interactions

© 2025 Dearborn LC et al. *JAMA Network Open*.

This figure represents the probability z-score of current asthma for a single pollutant when a second pollutant is held at various quantiles (a) and across pollutant concentrations (b). Pollutants on the y-axis in panel a were held at the 10th, 50th, and 90th quantile and represent the change in exposure-response function as the pollutant on the x-axis increases in concentration with shading indicating 95% credible intervals. Gaps in the credible intervals indicate portions where the corresponding bivariate concentrations were not observed. The relationship of interest is represented by the middle column where both NO_2 and $PM_{2.5}$ are held constant and O_3 changes in concentration. The predicted estimate of current asthma based on changing concentrations of two pollutants are represented in panel b, with red shading indicating higher predicted outcomes and blue shading indicating lower predicted outcomes.

© 2025 Dearborn LC et al. *JAMA Network Open*.

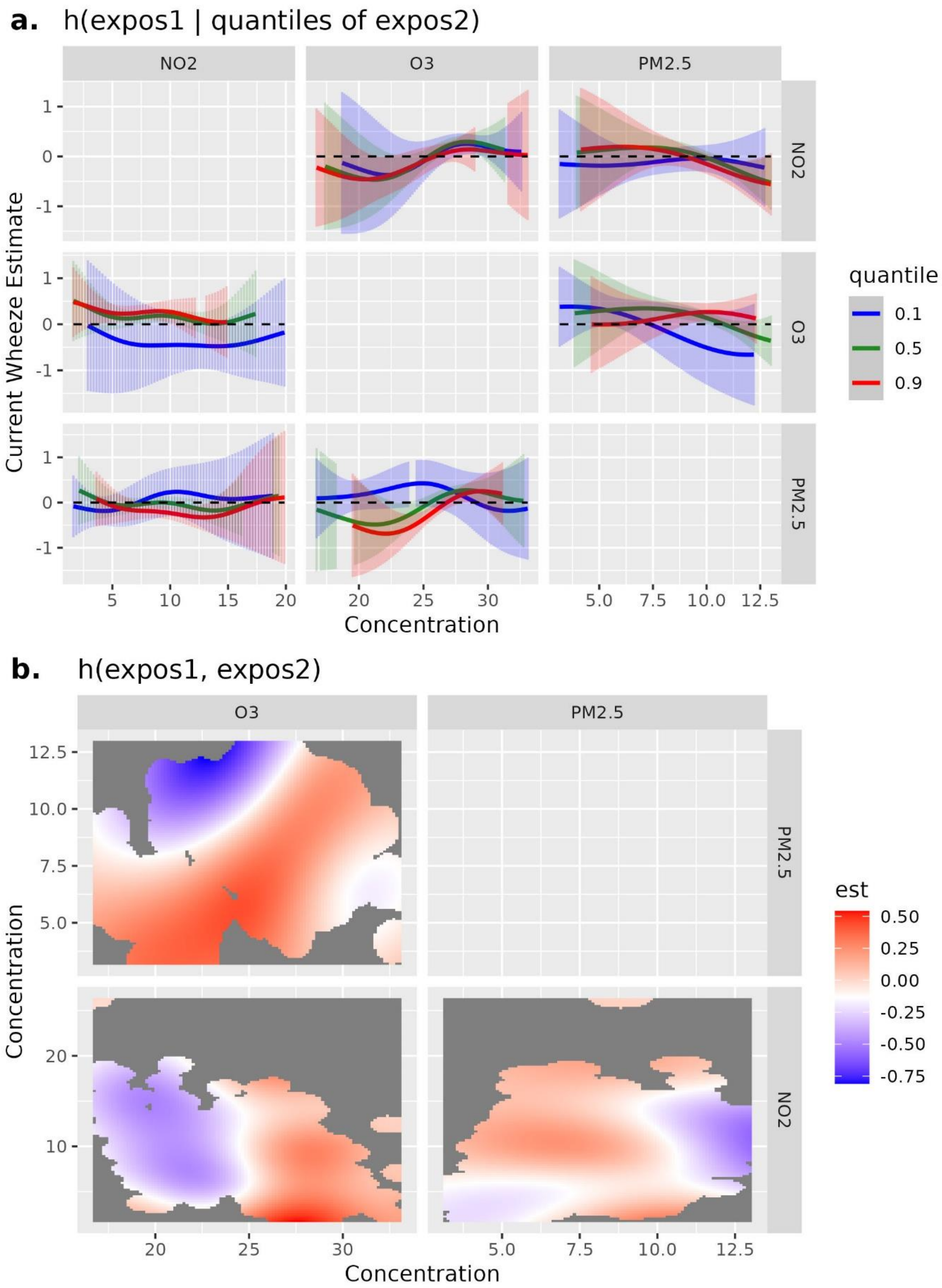

eFigure 7. Response function for the current wheeze outcome exploring bivariate pollutant interactions

© 2025 Dearborn LC et al. *JAMA Network Open*.

This figure represents the probability z-score of current wheeze for a single pollutant when a second pollutant is held at various quantiles (a) and across pollutant concentrations (b). Pollutants on the y-axis in panel a were held at the 10th, 50th, and 90th quantile and represent the change in exposure-response function as the pollutant on the x-axis increases in concentration with shading indicating 95% credible intervals. Gaps in the credible intervals indicate portions where the corresponding bivariate concentrations were not observed. The relationship of interest is represented by the middle column where both NO_2 and $PM_{2.5}$ are held constant and O_3 changes in concentration. The predicted estimate of current asthma based on changing concentrations of two pollutants are represented in panel b, with red shading indicating higher predicted outcomes and blue shading indicating lower predicted outcomes.

© 2025 Dearborn LC et al. *JAMA Network Open*.
