# Supplementary material for: Early-Life Ozone Exposure and Asthma and Wheeze in Children
Source: JAMA Netw Open. 2025 Apr 2;8(4):e254121. doi: 10.1001/jamanetworkopen.2025.4121 (PMC11966328; doi:10.1001/jamanetworkopen.2025.4121)
Supplement: Supplement 2. — Data Sharing Statement [file jamanetwopen-e254121-s002.pdf]

Data Sharing Statement

Dearborn. Early-Life Ozone Exposure and Asthma and Wheeze in Children. *JAMA Netw Open*. Published April 02, 2025. doi:10.1001/jamanetworkopen.2025.4121

Data
Data available: No

Additional Information
Explanation for why data not available: The data utilized for this study are not publicly available but de-identified data may be available on request, subject to approval by the internal review board and under a formal data use agreement. Contact the corresponding author for more information.
